# Efficacy of Intrauterine Bakri Balloon Tamponade in Cesarean Section for Placenta Previa Patients

**DOI:** 10.1371/journal.pone.0134282

**Published:** 2015-08-11

**Authors:** Hee Young Cho, Yong Won Park, Young Han Kim, Inkyung Jung, Ja-Young Kwon

**Affiliations:** 1 Division of Maternal-Fetal Medicine, Department of Obstetrics and Gynecology, Institute of Women’s Life Medical Science, Yonsei University College of Medicine, Yonsei University Health System, Seoul, Korea; 2 Department of Biostatistics, Yonsei University College of Medicine, Yonsei University Health System, Seoul, Korea; Kuopio University Hospital, FINLAND

## Abstract

**Purpose:**

The aims of this study were to analyze the predictive factors for the use of intrauterine balloon insertion and to evaluate the efficacy and factors affecting failure of uterine tamponade with a Bakri balloon during cesarean section for abnormal placentation.

**Methods:**

We reviewed the medical records of 137 patients who underwent elective cesarean section for placenta previa between July 2009 and March 2014. Cesarean section and Bakri balloon insertion were performed by a single qualified surgeon. The Bakri balloon was applied when blood loss during cesarean delivery exceeded 1,000 mL.

**Results:**

Sixty-four patients (46.7%) required uterine balloon tamponade during cesarean section due to postpartum bleeding from the lower uterine segment, of whom 50 (78.1%) had placenta previa totalis. The overall success rate was 75% (48/64) for placenta previa patients. Previous cesarean section history, anterior placenta, peripartum platelet count, and disseminated intravascular coagulopathy all significantly differed according to balloon success or failure (all *p*<0.05). The drainage amount over 1 hour was 500 mL (20–1200 mL) in the balloon failure group and 60 mL (5–500 mL) in the balloon success group (*p*<0.01).

**Conclusion:**

Intrauterine tamponade with a Bakri balloon is an adequate adjunct management for postpartum hemorrhage following cesarean section for placenta previa to preserve the uterus. This method is simple to apply, non-invasive, and inexpensive. However, possible factors related to failure of Bakri balloon tamponade for placenta previa patients such as prior cesarean section history, anterior placentation, thrombocytopenia, presence of DIC at the time of catheter insertion, and catheter drainage volume more than 500 mL within 1 hour of catheter placement should be recognized, and the next-line management should be prepared in advance.

## Introduction

Placenta previa occurs in approximately 4.8 of every 1,000 pregnancies [1] and is associated with maternal mortality and significant increase in maternal morbidities including massive hemorrhage, infection, adjacent organ damage, and emergency hysterectomy [2, 3]. Placenta previa-related uterine atony, bleeding from the lower flap of the uterine wall, and invasive placentation can cause postpartum hemorrhage (PPH) [4]. Intraoperative management options deployed to control hemorrhage in placenta previa patients include bimanual uterine compression, implantation site compression with sutures, uterine arterial ligation, pelvic arterial embolization, and hysterectomy.

Arterial ligation and compression suture have a low success rate among inexperienced surgeons, pelvic arterial embolization requires high medical costs and sophisticated facilities, and hysterectomy has high morbidity and mortality and confers fertility loss. Therefore, other non-invasive procedures are needed to treat PPH and preserve the uterus. In 1992, Bakri introduced intrauterine balloon tamponade for the treatment of obstetric hemorrhage during cesarean delivery [5]. A number of recent reports have described the successful use of balloon tamponade to manage hemorrhage from the lower uterine segment due to placenta previa-accreta. The overall success rate of balloon tamponade in controlling bleeding is reportedly 80% [6], but the heterogeneous causes of PPH, including uterine atony, retained placenta, genital tract laceration, and uterine rupture, are not specific for placenta previa [7].

The present study aimed to evaluate the outcomes of uterine tamponade using a Bakri balloon for PPH management in cases of placenta previa during caesarean deliveries and determine factors associated with its failure.

## Materials and Methods

### Study design and participants

This retrospective study assessed 137 patients who were diagnosed with placenta previa including a low-lying placenta and underwent elective cesarean section at the Severance Hospital from July 2009 to March 2014. The study protocol was approved by the institutional review board in Yonsei University Health System. Due to the retrospective nature of the study, informed consent was not necessary but patient records were de-identified prior to analysis.

Placenta previa was defined as a condition where the placenta lies low in the uterus, while partially or completely covering the cervix. The patients were diagnosed with placenta previa by 5–9-MHz transvaginal transducer using an iU22 ultrasound system (Philips Healthcare) in the third trimester. Placenta accreta is diagnosed using gray scale ultrasound and the suggestive signs of placenta accreta were presence of placental vascular lacunae, loss of a sonolucent area, interruption of bladder-uterine serosa and visualization of a focal protruding mass between the placenta and bladder. The diagnosis was reconfirmed within 1 week of elective cesarean section. Finally, suspected placenta accreta was diagnosed at the time of placental delivery during cesarean section. Presence of accreta was suspected when forced manual separation between chorionic plate and the myometrium was required due to firm placental attachment. No additional gross placental abnormalities, such as succenturiate lobe, vasa previa, or eccentric cord insertion, were identified in these cases.

### Interventions

All patients received general anesthesia for cesarean section. At the time of cesarean section, postpartum hemorrhage was initially managed with 20 IU of oxytocin infused in 1 L of 0.9% normal saline followed by intramuscular injection of 0.25 mg methylergometrine. In refractory cases, 0.1 mg of carbetocin was given intravenousely. Bakri balloon catheter insertion was performed in cases of more than 1000 mL postpartum hemorrhage and uncontrolled bleeding with uterotonics. Bakri balloon tamponade was not attempted in patients with chorioamnionitis, retained placenta, trauma of cervix and vagina, inherited coagulopathy and disseminated intravascular coagulopathy (DIC). We used the Bakri postpartum balloon (Cook Medical, Spencer, IN), which was the only device designed to control PPH available in Korea during this period [8]. The Bakri balloon tamponade was inserted through the cesarean section incision or transvaginally according to cervical opening. After proper placement of the catheter, the balloon was partially inflated with 50–100 mL of sterile normal saline. Then the assistant’s thumb and index finger were placed around the cervix at the level of internal os to keep the partially inflated balloon above the cervix. Uterine incision is closed in double-layer fashion using 1–0 absorbable suture material carefully avoid puncturing the balloon. Then, vaginal packing was placed using 3–10 tape gauze measuring 20 cm by 10 cm to prevent expulsion of the Bakri balloon. Following vaginal packing, balloon was further inflated up to 600 mL until the blood draining through catheter is significantly decreased. Throughout the procedure, the two fingers initially placed around the cervix were maintained to prevent drop of the balloon during vaginal packing placement or inflation. The total amount of tape gauze used for vaginal packing and total volume of normal saline used to inflate the balloon is recorded.

All patients were maintained on IV patient controlled analgesia for 48h to manage postsurgical pain. Pain was assessed every hour using Visual Analog Scale (VAS) and when VAS>7 or the patient opted for additional pain control, 25 mg pethidine was given IV or IM.

Post-balloon application, low-dose intravenous oxytocin infusion was maintained for 24 hours. The drainage amount was checked for hourly for the first 6 hours and if < 100 mL/h, every 4 h thereafter. The decision to remove the drain was made the following day. Criteria used for catheter removal were 1) drainage amount of < 50 mL/h and 2) serosanguineous drainage color. After removing the vaginal packing, the balloon was gradually deflated 100% over 1–5 min. The number of gauze tapes retrieved and the volume of normal saline withdrawn were crossed checked with the pre-balloon insertion note by 2 medical personnel. Patients were examined within 30 min of catheter removal for any sign of active bleeding. All patients received a single dose of cefazolin (1g, IV) within 30 min of surgical incision. Antibiotic was continued during balloon tamponade (cefazolin, 1g every 12 hours, IV) for 24 hours. All patients had a Foley catheter inserted into the bladder for absolute bed rest.

### Methods and Measurements

**Fig 1:**
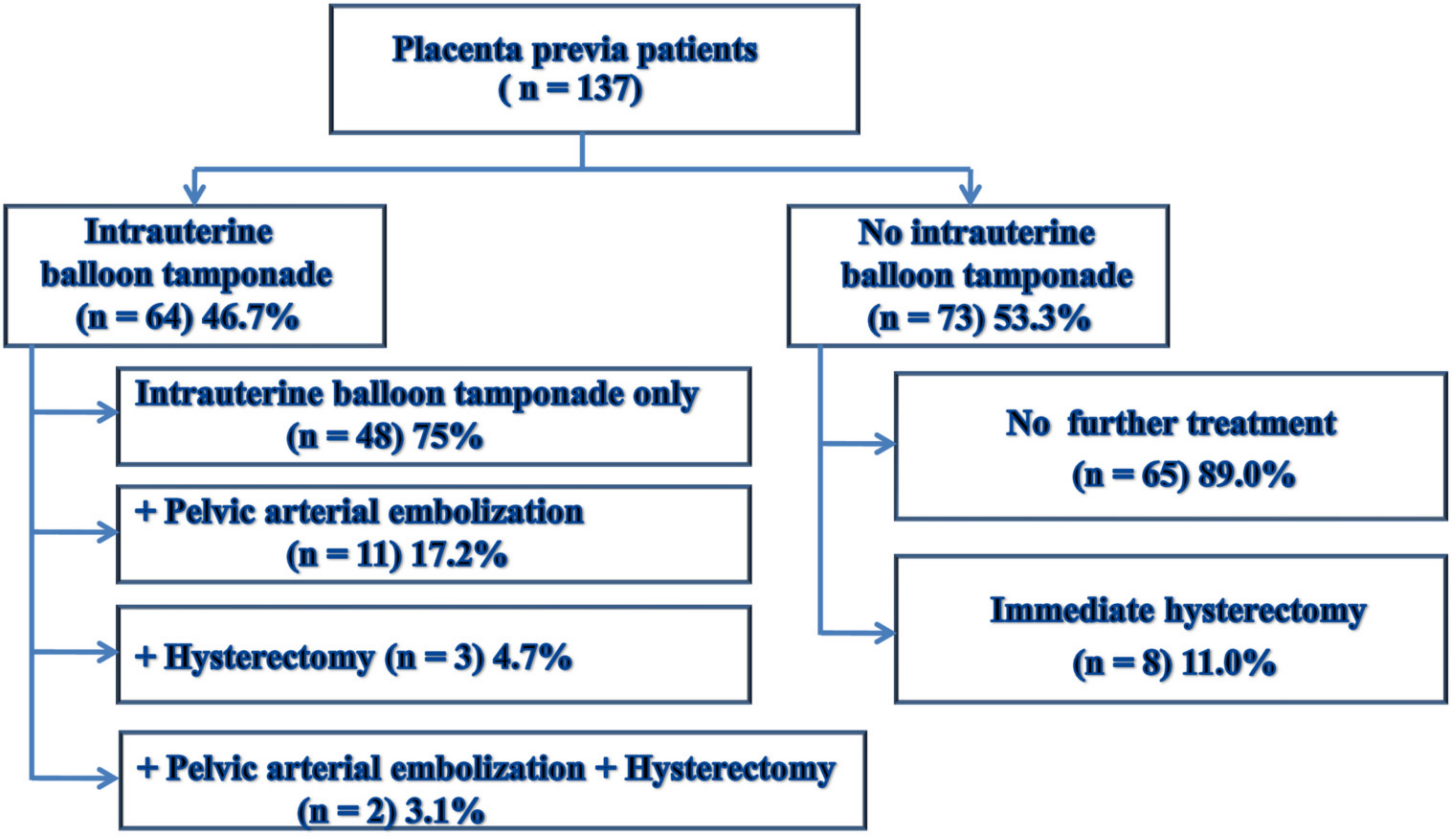
Management for Placenta Previa Patients.

The patients were divided into two groups, those with balloon tamponade insertion after caesarean delivery (balloon group, n = 64) and those without (non-balloon group, n = 73). To determine the utility of the balloon tamponade, the balloon tamponade group was further divided into a group in which only the balloon tamponade was inserted (n = 48) and a group in which the balloon tamponade was inserted and then pelvic arterial embolization (n = 11), cesarean hysterectomy (n = 3), or both of these interventions (n = 2) was performed. Among 73 patients in the non-balloon group, 65 underwent cesarean section only, and immediate hysterectomy was performed in 8. Eight patients in the non-balloon group with active bleeding, blood loss more than 10,000 mL, and a pulse rate more than 110 bpm during the surgery underwent immediate hysterectomy at the surgeon’s discretion and were excluded from further analysis (Fig 1). Therefore, the balloon group (n = 64) was compared with the non-balloon group excluding immediate hysterectomy cases (n = 65) in order to analyze the difference in each parameter between the balloon and non-balloon groups and to identify predictive factors of the use of intrauterine balloon insertion.

### Outcomes

We compared demographic and clinical characteristics including hemoglobin (g/dL), hematocrit (%), platelet count (10^3^/μL), total blood loss during surgery, red blood cell (RBC), platelet (PLT) transfusion status, postoperative vital signs, disseminated intravascular coagulopathy (DIC), and intensive care unit (ICU) admission between the balloon and non-balloon groups. Failure of Bakri balloon tamponade was defined as continuous uterine hemorrhage after proper placement and inflation of the balloon catheter, with the need for additional treatments to control the bleeding. The balloon tamponade group was thus further divided into a balloon success group, in which only the balloon tamponade was inserted (n = 48), and a balloon failure group (n = 16) in which balloon tamponade insertion was followed by pelvic arterial embolization (n = 11), cesarean hysterectomy (n = 3), or both (n = 2). In addition, we compared the balloon success group (n = 48) and balloon failure group (n = 16) to analyze the factors associated with Bakri balloon failure.

### Statistical analysis

For statistical processing, the Mann-Whitney *U* test or Fisher’s exact test was used for categorical variables, and two-sample *t*-test or Wilcoxon rank sum test was used for continuous variables. The analysis employed multivariate models of logistic regression that included obstetric characteristic factors. Statistical analysis was performed using SAS version 9.2 (SAS Institute, Inc., Cary, NC), and *p* values < 0.05 were considered statistically significant.

## Results

We investigated the medical records of 137 patients who were diagnosed with placenta previa (low-lying, 18; previa partialis and marginalis, 29; and previa totalis, 90) and underwent cesarean section. First, we compared patient demographic and clinical characteristics between the postpartum hemorrhage patients treated with Bakri balloon tamponade (balloon group) and non-balloon treated patients (non-balloon group) that underwent cesarean section only (Table 1).

**Table 1:** Demographic and Clinical Characteristics of the Women in the Uterine Balloon Tamponade and Non-balloon Tamponade Groups.

	Balloon group (n = 64)	Non-balloon group (n = 65)	*p* value
**Maternal age, years**	33.9±3.9	33.6±3.6	0.69
**BMI (kg/m** ^**2**^ **)**	25.2±3.3	25.4±3.1	0.73
**Gestational age, weeks**	36^+5^ [28^+0^–38^+0^]	36^+6^ [30^+0^–38^+0^]	0.17
**Primigravid**	35 (54.7)	37 (56.9)	0.19
**Multiple pregnancy**	3 (4.7)	2 (3.1)	0.68
**History of prior delivery**			
Vaginal delivery (≥1)	11 (17.2)	16 (24.6)	0.49
Cesarean section (≥1)	17 (26.6)	12 (18.5)	0.30
**History of D&E (≥1)**	17 (26.6)	20 (30.7)	0.56
**Uterine abnormal findings**			0.63
None	58 (90.6)	56 (86.1)	
Adenomyosis	1 (1.6)	2 (3.1)	
Myoma	4 (6.3)	5 (7.7)	
Other	1 (1.5)	2 (3.1)	
**Type of previa**			<0.01
Totalis	50 (78.1)	33 (50.8)	
Partialis, marginalis	11 (17.2)	18 (27.7)	
Low-lying placenta	3 (4.7)	14 (21.5)	
**Placental location**			0.32
Anterior	8 (12.4)	11 (17.0)	
Posterior	52 (81.3)	53 (81.5)	
Lateral	4 (6.3)	1 (1.5)	
**Presence of placenta accreta**	19 (30.0)	4 (6.2)	<0.01

Data in the table are presented as n (%), mean ± SD, and median [range].

The balloon and non-balloon groups did not significantly differ in terms of their maternal age, gestational age at delivery, parity, or medical history such as previous vaginal or cesarean delivery, history of dilatation & evacuation (D&E), or abnormal uterine findings. The prevalence of patients with placenta previa totalis and placenta accreta was significantly higher in the balloon group than in the non-balloon group. The frequency of placenta previa totalis was 78.1% and 50.8% of patients in the balloon and non-balloon groups, respectively (*p<*0.01). The occurrence of placenta accreta was 30.0% in the balloon group and 6.2% in the non-balloon group (*p*<0.01) (Table 1).

Mean total blood loss during surgery was 1769 mL (600–6900 mL) in the balloon group and 725 mL (400–2200 mL) in the non-balloon group (*p*<0.01) (Table 2). Although indication for balloon insertion was blood loss >1000 mL, one case with only 600 ml hemorrhage was also treated with balloon tamponade. This case was completely non-responsive to conventional uterotonics, bleeding progressed over a very short time (30 min), and pulse rate measured greater than 120 BPM. Blood transfusion during surgery was reported in 52 cases (81.0%) in the balloon group and 11 cases (16.9%) in the non-balloon group (*p*<0.01), however total volume transfused was not significantly different between groups (data not shown). Peripartum pulse rate, peripartum PLT, frequency of ICU admission, and days of hospital stay also significantly differed between the balloon and non-balloon groups (*p*<0.01).

**Table 2:** Comparison of Measures of Severity and Outcomes between Uterine Balloon Tamponade and Non-balloon Tamponade Groups.

	Balloon group (n = 64)	Non-balloon group (n = 65)	*p* value
**Estimated blood loss, mL**	1769 [600–6900]	725 [400–2200]	<0.01
**RBC transfusion**	52 (81.0)	11 (16.9)	<0.01
**PLT transfusion**	3 (5.0)	0 (0)	0.13
**Peripartum systolic BP, mmHg**	118.7±16.6	117.7±16.6	0.71
**Peripartum diastolic BP, mmHg**	69.4±12.1	67.1±12.4	0.29
**Peripartum pulse rate, /min**	84.9±13.0	77.5±11.2	<0.01
**Peripartum PLT, 10** ^**3**^ **/μL**	173.4±67.0	227.2±64.8	<0.01
**Disseminated intravascular coagulopathy**	4 (6.0)	0 (0)	0.05
**Intensive care unit admission**	11 (17.0)	0 (0)	<0.01
**Length of hospital stay, days**	6 [3–56]	5 [3–18]	<0.01

Data in the table are presented as n (%), mean± SD, and median [range].

PLT, platelet; RBC, red blood cell; PT, prothrombin time; PTT, partial thromboplastin time; BP, blood pressure

Of the 64 patients who underwent Bakri balloon tamponade, 48 patients (75%) were treated with only this procedure (balloon success group), and the remaining 16 patients (25%) underwent pelvic arterial embolization or hysterectomy or both after Bakri balloon trial (balloon failure group). Therefore, we compared the balloon success and failure groups to investigate factors associated with balloon tamponade failure (Table 3). In preoperative laboratory results, although within normal range, the values of PLT, prothrombin time (PT), and partial thromboplastin time (PTT), were statistically different between the success and failure groups. Lower preoperative PLT values (*p* = 0.02), and higher PT and PTT values (*p* = 0.01, *p*<0.01 respectively) were observed in the balloon failure group. The total operative blood loss in the balloon success group was 1,502 mL (600–4,520 mL), and that in the balloon failure group was 3,180 mL (1,220–6,900 mL, *p* = 0<0.01). Consequently, the RBC, PLT transfusion rate, pain scale, peripartum pulse rate, peripartum PLT, DIC, frequency of ICU admission, and days of hospital stay also significantly differed between the two groups. The drainage amount over 1 hour was 500 mL (20–1200 mL) in the balloon failure group and 60 mL (5–500 mL) in the balloon success group (*p*<0.01).

**Table 3:** Comparison of Measures of Severity and Outcomes between Uterine Balloon Tamponade Success and Balloon Failure Groups.

	Balloon success group (n = 48)	Balloon fail group (n = 16)	*p* value
**Preop hemoglobin, g/dL**	11.2±1.3	10.9±1.6	0.87
**Preop hematocrit, %**	33.2±4.0	32.2±5.0	0.67
**Preop platelet, 10** ^**3**^ **/μL**	216.8±54.0	175.1±54.4	0.02
**Preop platelet < 100,000/μL**	1 (2.1%)	2 (12.5%)	0.15
**Prothrombin time, s**	10.1 [8.8–14.4]	11.1 [9.0–22.4]	0.01
**Partial thromboplastin time, s**	26.4 [21.0–46.1]	30.5 [24.4–67.9]	<0.01
**Red blood cell transfusion**	36 (75.0%)	16 (100%)	0.03
**Platelet transfusion**	0 (0%)	3 (18.8%)	0.01
**Estimated blood loss, mL**	1502 [600–4520]	3180 [1220–6900]	<0.01
**Peripartum systolic BP, mmHg**	120.0±17.6	114.9±13.2	0.32
**Peripartum diastolic BP, mmHg**	69.3±12.5	69.6±11.3	0.94
**Peripartum pulse rate, /min**	77.5±11.2	85.4±13.1	<0.01
**Peripartum platelet, 10** ^**3**^ **/μL**	192.7±60.2	115.4±52.2	<0.01
**Peripartum platelet < 100,000/μL**	3 (6.2%)	8 (50.0%)	<0.01
**Disseminated intravascular coagulopathy**	0 (0%)	4 (25.0%)	<0.01
**Uterine balloon volume, mL**	200 [100–500]	275 [150–600]	0.01
**Vaginal packing**	46 (95.8)	13 (100)	1.00
**Drainage amount in 1 h**	60 [5–500]	500 [20–1200]	<0.01
**Intensive care unit admission**	1 (2.1%)	10 (62.5%)	<0.01
Length **of hospital stay, days**	5 [3–31]	7 [5–56]	<0.01

Data in the table are presented as n (%), mean ± SD, and median (range).

Preop, preoperative; BP, blood pressure

Nulliparous women were less frequent (*p* = 0.01) and women with a previous history of cesarean section were more frequent (*p*<0.01) in the balloon failure group than in the balloon success group, and the number of cases of anterior placenta (*p*<0.01) and placenta accreta (*p* = 0.04) significantly differed between the two groups. In addition, the rates of less than 3-mm lower uterine segment flap thickness were 16.7% and 43.8% in the balloon success and failure groups, respectively (*p* = 0.02) (Table 4). However, multiple regression analysis indicated that Bakri balloon tamponade failure showed significant association only with anterior placenta (OR, 12.75, CI 1.04–155.94) and history of cesarean section (OR, 8.90, CI 2.27–34.83) among obstetric characteristics (Table 5).

**Table 4:** Comparison of General and Obstetric Characteristics between Uterine Balloon Tamponade Success and Failure Groups.

	Balloon success group (n = 48)	Balloon fail group (n = 16)	*p* value
**Maternal age, years**	33.4±4.2	35.4±2.8	0.06
**Gestational age, weeks**	36^+5^ [32^+0^–38^+0^]	36^+6^ [28^+0^–38^+0^]	0.30
**Parity**			0.01
Nulliparous	31 (64.6)	4 (25.0)	
1	14 (29.2)	8 (50.0)	
≥2	3 (6.2)	4 (25.0)	
**Multiple pregnancy**	1 (2.1)	2 (12.5)	0.15
**History of delivery**			
Vaginal delivery(≥1)	9 (18.7)	2 (12.5)	0.34
Cesarean section(≥1)	7 (14.6)	10 (62.5)	<0.01
**History of D&E (≥1)**	13 (27.1)	4 (15)	0.40
**Uterine abnormal findings**			1.00
None	43 (89.6)	15 (93.8)	
Adenomyosis	1 (2.1)	0 (0)	
Myoma	3 (6.2)	1 (6.2)	
Other	1 (2.1)	0 (0)	
**Types of previa**			0.63
Totalis	36 (75.0)	14 (87.5)	
Partialis, marginalis	9 (18.8)	2 (12.5)	
Low-lying placenta	3 (6.2)	0 (0)	
**Placental location**			<0.01
Anterior	2 (4.2)	6 (37.5)	
Posterior	42 (87.5)	10 (62.5)	
Lateral	4 (8.3)	0 (0)	
**Presence of placenta accreta**	11 (22.9)	8 (50.0)	0.04
**Lower uterine segment flap thickness < 3 mm**	8 (16.7)	7 (43.8)	0.02
**Direction of insertion**			0.10
Incision → vagina	26 (54.2)	11 (78.6)	
Vagina → incision	22 (45.8)	3 (21.4)	

Data in the table are presented as n (%), mean ± SD, and median [range].

**Table 5:** Multiple regression analysis of general and obstetric characteristics between Bakri balloon success and failure group.

Variable	OR	95% CI	*p* value
**Anterior placenta**	12.75	1.04–155.94	0.04
**History of cesarean section**	8.90	2.27–34.83	<0.01

OR, odds ratio; CI, confidence interval.

## Discussion

This study on the Bakri balloon catheter is the largest published report to evaluate a single type of uterine tamponade device for the treatment of PPH due to placenta previa. In addition, this report is the first study to statistically analyze reasons for failure of Bakri balloon tamponade. Our findings are as follows: prior history of one of more cesarean section, anterior placentation, thrombocytopenia, presence of DIC at catheter insertion, and catheter drainage volume more than 500 mL within 1 hour of catheter placement were associated with Bakri balloon tamponade failure to control PPH.

PPH is blamed for more than 30% of maternal deaths, but this rate can be reduced through appropriate prevention, diagnosis, and management [2, 9]. First-line treatments for PPH include uterotonic drugs and bimanual compression. The second-line treatment is uterine compression suture, internal iliac artery ligation, or pelvic arterial embolization. However, pelvic arterial embolization cannot be performed promptly in the operating room because the cesarean section incision must first be closed, and it requires a radiology intervention suite. Therefore, if massive bleeding persists, caesarean hysterectomy must be performed to avoid maternal mortality [10–13]. By contrast, intrauterine tamponade can be used immediately in the operating room and increase the chance of preserving fertility.

Previous studies have reported the benefit of balloon tamponade for massive PPH regardless of cause and suggested that balloon tamponade should be part of all protocols in the management of PPH [14–16]. The outcomes of this study indicate that Bakri balloon tamponade can be used when initial bleeding is not controlled by medical treatments and is useful for cases of placenta previa totalis and massive hemorrhage. We have used the Bakri balloon tamponade since 2009, because it was the only catheter approved for use to control PPH that was available in Korea. Doumouchtsis et al. [17] evaluated the success rates of several management methods for PPH and found an 84% success rate of balloon tamponade. More recently, Laas et al. [18] reported an 86% global success rate of uterine balloon tamponade in a before-and-after study to evaluate its utility for PPH management. The present study is the first to report the success rate of Bakri balloon tamponade specifically in placenta previa patients, which reached 75% (48/64 women). In our study, the rate of balloon displacement was 4.7% (3/64 women) which is lower than what other studies described (6–16%) [19]. Based on our experience, the balloon displacement usually occurs at the time of inflation and/or immediately after inflation due to following reasons: 1) incorrect placement of the balloon catheter by inexperienced operator, 2) inability of the dilated cervix to support the balloon, and/or 3) inadequate vaginal packing. Our relative success may be attributed to the placement of balloon and vaginal packing by a single expert and the characteristic of patients included in this study being non-labored elective cesarean section at earlier gestation, 54.7% primigravid, and 36.8% of patients with unripened cervix.

One previous study reported that problems of balloon catheterization included obstruction by uterine myoma, unintentional damage of the catheter, and inability to use with other treatments simultaneously [20]. The same study associated balloon tamponade failure with placenta previa with percreta and DIC. Consistently, we found several factors that were associated with failure of the Bakri balloon tamponade. In multiple regression analysis, Bakri balloon tamponade failure was associated with anterior placenta (OR, 12.75, CI 1.04–155.94) and history of cesarean section (OR, 8.90, CI 2.27–34.83) among obstetric characteristics. Regarding the etiology of these associations, we speculate this might be based upon the increased incidence of placenta accreta in women with history of prior cesarean delivery, and the documented association between anterior placentation and increased hemorrhage. However, the results should be interpreted with caution since confidence intervals were very wide due to small number cases in the Bakri failure group.

The American College of Obstetrics and Gynecology (ACOG) report a rate of accreta of 40% in patients with a history of two prior cesarean sections and anterior or central placenta previa [21]. Another study showed that incidence of massive blood loss, massive transfusion, placenta accreta and hysterectomy were specifically higher in placenta previa patients with anteriorly located implantation sites [22].

Our study also showed that if drainage from the tube exceeds 500 mL within 1 hour of Bakri balloon catheter insertion, the treatment should be considered a failure, and the next step in management should be initiated immediately. In the balloon group, we suspected 19 cases to involve accreta and 11 of these patients were successfully treated by Bakri balloon tamponade without any further invasive procedures. Of the eight patients with suspected accreta and balloon failure, five who underwent cesarean hysterectomy had pathologically confirmed placenta accreta and increta. Therefore, whether balloon tamponade should be recommended in cases of severe attachment of the placenta to the uterus requires further investigation.

In addition, the present study raises several practical considerations for intrauterine balloon catheterization such as the direction of balloon catheter insertion depending on cervical dilatation, range of uterine balloon volume, vaginal packing after the procedure, and duration of balloon tamponade. A minimum of 100 mL and a maximum 600 mL of inflation of the balloon is necessary to manage hemorrhage in cesarean section patients with placenta previa. As the diameter of inflation port of the catheter is approximately 2 cm, transabdominal placement of the catheter (passing the inflation port of the catheter into the uterine incision and through the cervix) is possible only when the cervical opening evaluated at the time of catheter insertion is more than 2 cm. If the cervical opening is less than 2 cm, transvaginal approach should be opted. A routine vaginal packing placement is recommended because previously, we experienced few cases of balloon displacement within 2 hours of insertion that ended up in heavy hemorrhaging either in the recovery room or on ward. We noted that their cervix was dilated to 4–5 cm at the time of expulsion. Thus, our speculation is that Bakri balloon acted similar to Foley’s catheter balloon used for cervical ripening. So following these unfortunate experiences, we implemented routine vaginal packing.

We acknowledge the limitations of this study. Firstly, as our study design was a non-randomized retrospective study from a single-center, our data cannot draw conclusion on the efficacy of intrauterine balloon tamponade in comparison to other treatment modalities. The intent of the present study was to report on the experience on the application of Bakri balloon catheter in the management of hemorrhage encountered during cesarean section for placenta previa. Secondly, the diagnosis of PPH can be very subjective as accurate measurement of blood shed is impossible. This will always remain the limitation to studies in the context of hemorrhage.

Future studies should address which type of balloon catheter is most effective for the management of PPH, because few studies have compared the efficacy of balloon tamponade devices. Additional research should also address short and long-term outcomes such as uterine rupture, Asherman syndrome, resumption of menses and success rate of subsequent pregnancy. A randomized controlled trial and multi-center study to compare intrauterine balloon tamponade with embolization, compressive sutures and uterine artery ligation should be conducted.

In conclusion, Bakri balloon tamponade is effective, simple to deploy, allows rapid placement, and can provide immediate results with minimal complications for PPH. Intrauterine balloon tamponade should be considered as the second-line treatment in massive hemorrhages that are unresponsive to uterotonics. Our data demonstrate factors affecting failure of Bakri balloon tamponade; therefore, next-line treatments should be anticipated for placenta previa patients with these factors.
